# Phantom-based evaluation of yttrium-90 datasets using biograph vision quadra

**DOI:** 10.1007/s00259-022-06074-3

**Published:** 2022-12-12

**Authors:** Konstantinos G. Zeimpekis, Lorenzo Mercolli, Maurizio Conti, Hasan Sari, George Prenosil, Kuangyu Shi, Axel Rominger

**Affiliations:** 1 Department of Nuclear Medicine, Inselspital, Bern University Hospital, University of Bern, Bern, Switzerland; 2 Molecular Imaging, Siemens Healthineers, Knoxville, TN USA; 3 Advanced Clinical Imaging Technology, Siemens Healthcare AG, Lausanne, Switzerland

**Keywords:** Whole-body PET/CT, Yttrium-90, NEMA, Image quality, Quadra

## Abstract

**Purpose:**

The image quality characteristics of two NEMA phantoms with yttrium-90 (^90^Y) were evaluated on a long axial field-of-view (AFOV) PET/CT. The purpose was to identify the optimized reconstruction setup for the imaging of patients with hepatocellular carcinoma after ^90^Y radioembolization.

**Methods:**

Two NEMA phantoms were used, where one had a 1:10 sphere to background activity concentration ratio and the second had cold background. Reconstruction parameters used are as follows: iterations 2 to 8, Gaussian filter 2- to 6-mm full-width-at-half-maximum, reconstruction matrices 440 × 440 and 220 × 220, high sensitivity (HS), and ultra-high sensitivity (UHS) modes. 50-, 40-, 30-, 20-, 10-, and 5-min acquisitions were reconstructed. The measurements included recovery coefficients (RC), signal-to-noise ratio (SNR), background variability, and lung error which measures the residual error in the corrections. Patient data were reconstructed with 20-, 10-, 5-, and 1-min time frames and evaluated in terms of SNR.

**Results:**

The RC for the hot phantom was 0.36, 0.45, 0.53, 0.63, 0.68, and 0.84 for the spheres with diameters of 10, 13, 17, 22, 28, and 37 mm, respectively, for UHS 2 iterations, a 220 × 220 matrix, and 50-min acquisition. The RC values did not differ with acquisition times down to 20 min. The SNR was the highest for 2 iterations, measured 11.7, 16.6, 17.6, 19.4, 21.9, and 27.7 while the background variability was the lowest (27.59, 27.08, 27.36, 26.44, 30.11, and 33.51%). The lung error was 18%. For the patient dataset, the SNR was 19%, 20%, 24%, and 31% higher for 2 iterations compared to 4 iterations for 20-, 10-, 5-, and 1-min time frames, respectively.

**Conclusions:**

This study evaluates the NEMA image quality of a long AFOV PET/CT scanner with ^90^Y. It provides high RC for the smallest sphere compared to other standard AFOV scanners at shorter scan times. The maximum patient SNR was for 2 iterations, 20 min, while 5 min delivers images with acceptable SNR.

## Introduction

The procedure of radioembolization with yttrium-90 (^90^Y) has been increasingly used for treating patients with malignant liver tumors such as hepatocellular carcinoma (HCC) [1–5]. ^90^Y radioembolization, also known as selective internal radiation therapy (SIRT), utilizes glass or resin microspheres containing ^90^Y. The spheres can irradiate tumor tissue by locally depositing their energy without significantly affecting the surrounding healthy tissue in the liver. The administration is performed via selected branches of the hepatic artery.

Single-photon emission computed tomography (SPECT) has been regarded the standard for the post-treatment dosimetry validation [6, 7]. However, the ^90^Y Bremsstrahlung SPECT imaging presents certain challenges [6, 8, 9] such as dominant photon scatter, collimator septal penetration, and limited spatial resolution [10].

Apart for the predominantly emitted beta particles, a rare branch of beta minus of ^90^Y in its disintegration goes to the first excited state of zirconium-90 (^90^Zr) where the origin of the beta plus can be explained following a rare monopole transition between the 0 + /0 − states of ^90^Zr resulting in an internal pair production [11]. Modern positron emission tomography with computed tomography scanners (PET/CTs) can overcome the limitations arising from the low count statistics of the extraordinary small branching ratio (32 ppm) [12] based on their overall higher sensitivity performance. Specifically, for the post-treatment dosimetry evaluation, which is important for assessing the outcome of the irradiation of the tumor lesions, PET has not only been gaining favor but it is strongly recommended for ^90^Y resin microsphere therapy [13] due to its higher spatial resolution and higher quantitative accuracy compared to SPECT [6, 8, 9, 14–16]. The increased use of PET for SIRT imaging has been made possible by the introduction of TOF PET (12, 13), which reduces the noise and serves as an effective sensitivity increaser: such an effect is known to be directly connected to the time resolution of PET scanner [17].

With the recent introduction of long axial field-of-view (LAFOV) scanners, such as the Biograph Siemens Vision Quadra and the uEXPLORER, the sensitivity has increased multiple times compared to the standard axial field-of-view (AFOV) scanners. The peak sensitivity for the Biograph Vision Quadra at the center of the AFOV is 176 cps/kBq which is 2.75 times higher than the Biograph Vision 600 which has a standard AFOV of 26.3 cm [18]. The uEXPLORER with an AFOV of 196 cm provides a peak sensitivity at 174 cps/kBq [19]. In addition, silicon photomultipliers (SiPMs) that have replaced the bulky photomultiplier tubes provide higher overall gain, energy, and timing resolution that further enhance the scanner’s sensitivity.

However, the Biograph Vision Quadra has similar timing and spatial resolution to the Biograph Vision 600 since they both use SiPM detectors based on same technology [18]. A study has already evaluated National Electrical Manufacturers Association (NEMA) body phantom [20] datasets acquired on an uEXPLORER and has shown that the SNR for the smaller spheres, with diameters 13 mm and 10 mm, was higher compared to Biograph mCT PET/CT which has an AFOV of 21.8 cm and a sensitivity of 9.6 cps/kBq [21]. In this work, ^90^Y datasets of two NEMA body phantoms, acquired on a LAFOV PET/CT scanner with an AFOV of 106 cm, were reconstructed with different parameters as well as acquisition times and evaluated in terms of image quality metrics, like mean contrast recovery coefficient (RC_mean_) and signal-to-noise ratio (SNR). Taking advantage of the higher overall sensitivity of the scanner compared to scanners with standard AFOV (15–30 cm), the purpose is to define the optimal reconstruction setup that provides maximum SNR and high RC_mean_ as well as minimizing scan time for patient comfort.

## Materials and methods

### Scanner

A Biograph Vision Quadra PET/CT scanner (Siemens Healthineers, Knoxville, TN, USA) was used which employs silicon photomultiplier-based detectors with 3.2 × 3.2 × 20.0 mm lutetium-oxoorthosilicate crystals [18]. The Biograph Vision Quadra has 32 detector rings, each one with 38 detector blocks, which provide an AFOV of 106 cm. The data were acquired using the complete AFOV, in ultra-high sensitivity (UHS) mode, with the maximum ring distance (MRD) of 322 crystals (MRD 322). In addition to this UHS mode, images were also reconstructed using the high sensitivity (HS) mode that was the default reconstruction before UHS was available, with a maximum ring distance of only 85 crystals (MRD 85) [18].

Specifically, the Biograph Vision Quadra records all possible lines of response (LORs) using MRD 322, with an acceptance angle of 52 degrees. In the first version of the reconstruction software (VR10), also named high-sensitivity mode, images are reconstructed with LORs spanning an MRD of 85 crystal rings (MRD 85). This MRD is comparable to the Biograph Vision 600 MRD of 79 [22], corresponding to an acceptance angle for axial LOR of about 18°. The MRD metric refers to the number of crystals in the LOR’s axial extent and includes the gaps between blocks. In MRD 85 mode, the Vision Quadra does not use all the possible LORs between scintillating crystals for image reconstruction. In short, UHS has maximum ring difference of 322; HS has maximum ring difference of 85. UHS has acceptance angle of 54 degrees whereas HS has 18 degrees. The data is always acquired with full ring difference; HS and UHS are just different reconstructions [18].

The overall system sensitivity is 176 cps/kBq for UHS and 83 cps/kBq for HS mode, while the time-of-flight (TOF) is 230 ps and 228 ps, respectively [18]. Furthermore, an improved 3D scatter correction algorithm is utilized, where the full 3D scatter profile is estimated based on the residuals between measured and modeled data [23]. In this novel approach, the 2D measured data along with the 2D single scatter simulation (SSS) model-based scatter was used to provide the non-scattered true image estimate. Since the single and multiple scatter in oblique angles for LAFOV scanners is more prominent, the scatter for these segments was computed as the residual between the measured net trues and the non-scattered true estimate.

Attenuation correction was based on the CT data.

### Phantom preparation

Two NEMA International Electrotechnical Commission (IEC) body phantoms (Data Spectrum Corp.) were used, with 6 fillable spheres with inner diameters of 10, 13, 17, 22, 28, and 37 mm [20]. The background volume of one of the phantoms was filled with distilled water and activity (hot phantom) while the other one was filled with non-radioactive distilled water (cold phantom). The background volumes of both phantoms (9829 ml and 9762 ml, respectively) were weighted with the lung and sphere inserts placed inside the phantoms. The first phantom was filled at ~ 1:10 of its whole volume capacity plus twice the volume of the spheres inserts, and the whole activity was infused in that volume. After shaking the phantom for homogeneity, another syringe was used to extract enough volume from the background to fill the spheres inserts of both phantoms. For the purposes of our study, liquid [^90^Y]-yttrium citrate, in 0.5 M hydrochloric acid to prevent adhesion to the plastic phantom walls, was used for convenience, to fill the spheres and the background of the hot phantom, instead of microspheres. The activity was measured with a well-type dose calibrator (ISOMED 2010). The calibrator satisfies the Swiss regulatory requirements and was calibrated by the Swiss Federal Institute of Metrology (METAS) [24]. The drawn activity, by an automatic injector, was measured 1309.48 MBq while the residual activity in the syringe was 0.1 MBq. Therefore, the background activity concentration of the hot phantom was ~ 0.13 MBq/ml and the spheres’ activity concentration for both phantoms was ~ 1.3 MBq/ml. The phantom was filled the day before the scan and left to decay to represent the average injected activities at the clinic of around 1 GBq (background and spheres’ activity concentration at 0.1 and 1.0 MBq/ml, respectively at the time of the scan).

### Scan

Both phantoms were positioned on the bed around the center with a 10-cm gap between them. A helical CT scan was acquired for the PET attenuation correction with the following parameters: 80 kV tube voltage, 39 modulated mAs tube current, 38.4 mm total collimation width, 5 mm slice thickness, and a pitch 0.8. The reconstruction matrix for the CT images was 512 × 512 with 644 slices, and the voxel dimensions were 1.523 × 1.523 × 1.600 mm^3^. Following the CT, the PET acquisition was performed with a total acquisition time of 50 min. The AFOV covered both phantoms. The voxel dimensions were 3.30 × 3.30 × 1.65 mm^3^. The scanner had been calibrated before for ^90^Y and a calibration factor was used for normalizing the data. The data were decay corrected to the injection time.

### Reconstruction

All of the data were reconstructed with TOF, point spread function (PSF) recovery, and ordered subset expectation maximization (OSEM). Then, the list-mode data were reconstructed with different parameters. The images were generated with iteration numbers from 2 to 8 with 5 subsets, a Gaussian filter of 2–6 mm full width at half maximum (FWHM), a matrix size of 440 × 440 and 220 × 220, and reconstructions with HS and UHS. Furthermore, images, except for the full-data 50-min acquisition, were generated using the first 40, 30, 20, 10, and 5 min of the list-mode data. In total, there were 840 reconstructed image datasets (Table 1). To test the performance of the product of iterations and subsets on RC_mean_ at low count statistics, the phantom data for the 5-min acquisition were also reconstructed with 10 iterations and 1 subset.

**Table 1:** Summary of all the parameters used to reconstruct the original dataset

Mode	HS UHS
Reconstruction matrix	220 × 220 440 × 440
Number of iterations	2, 3, 4, 5, 6, 7, 8
Number of subsets	5
Gaussian filter FWHM (mm)	2, 3, 4, 5,6
Reconstructed times (min)	05, 10, 20, 30, 40, 50

### Analysis

The analysis was performed for both hot and cold phantoms. Volume-of-interest (VOI) masks were manually drawn on both phantom PET images using itk-SNAP, an open-source software [25], centered on the slice where the smallest sphere was best visualized. These included spherical VOIs over the 6 spheres, 10 background VOIs with the same size as the biggest sphere VOI, 5 background VOIs with similar sizes to the smaller spheres, and a VOI placed on the lung insert with a diameter size of 30 mm. The performance characteristic analysis includes the RC_mean_ for all fillable spheres, lung error, i.e., the percentage counts in the lung, background variability [26], and signal-to-noise ratio. Similar to the NEMA standard, but using VOIs instead of regions-of-interest (ROIs), the RC_mean_ was defined as:

$${\mathrm{RC}}_{\mathrm{mean}}=\frac{\left(\frac{{\text{S}}\mathrm{s,j}}{{\text{S}}\mathrm{b,j}}\right)\text{-1}}{\left(\frac{{\text{a}}\mathrm{s,j}}{{\text{a}}\mathrm{b,j}}\right)\text{-1}}$$

where *S*_*S,j*_ is the average counts in the VOI for sphere *j*, *S*_*b,j*_ is the average of the background VOI counts for sphere *j*, *a*_*s,j*_ is the activity concentration in the hot spheres, and *a*_*b,j*_ is the activity concentration in the background.

For the cold phantom, the recovery coefficients of the spheres were calculated as before, without normalization to the background concentration. The SNR was calculated as the ratio of the measured VOI mean signal of each sphere to the corresponding background VOI standard deviation.

The percent background variability for each sphere was calculated as the ratio of the spheres’ VOI standard deviation to the background VOI mean signal. The lung error was calculated as the ratio of the lung insert VOI mean signal to the average of the ten 37-mm-diameter background VOI mean signals. Image viewing was performed on syngo.via (Siemens Healthineers, Chicago, IL, USA).

### Patient data

One male patient of 70 years old, diagnosed with HCC and pulmonary metastatic colorectal cancer injected with 1.1 GBq activity, received a PET scan on a Biograph Vision Quadra after ^90^Y resin microsphere radioembolization. The images were reconstructed using the clinical protocol with 4 iterations and 5 subsets, a 2-mm FWHM Gaussian filter, a 440 × 440 reconstruction matrix, and an acquisition time of 20 min. Data were reconstructed also with 2 iterations and a 220 × 220 reconstruction matrix, based on the phantom data evaluation. The list-mode data were also rebinned and reconstructed using 1-, 5-, and 10-min frames. The SNR was defined as the mean VOI signal of the lesion with the highest uptake concentration to the liver’s background VOI standard deviation. The coefficient of variation (COV) which is the background variation was defined as the ratio of the liver’s background VOI standard deviation to the liver’s background VOI mean signal.

### Statistical analysis

The uncertainties of the RC_mean_ results were based on the standard deviation of the mean. The statistical significance of the difference between the results was performed using the two-tailed paired *t*-test with a *p* value < 0.05.

## Results

Due to the huge number of reconstructed datasets, only the results regarding the clinically optimum trade-off between the RC_mean_ and SNR values are listed. A summary of the maximum and minimum values can be found in Table 2. All other results can be seen on the figures.

**Table 2:** Recovery coefficients, background variability, and lung error for both phantoms for 2 and 8 iterations

UHS 2-mm Gaussian FWHM	RC_mean_						Background variability %						
	37 mm	28 mm	22 mm	17 mm	13 mm	10 mm	37 mm	28 mm	22 mm	17 mm	13 mm	10 mm	Lung error %
*Hot phantom*													
2 iterations													
50 min	0.84	0.68	0.63	0.53	0.45	0.36	27	27	27	26	30	33	18%
40 min	0.85	0.68	0.62	0.52	0.48	0.35	30	30	29	32	25	33	16%
30 min	0.85	0.72	0.62	0.57	0.45	0.34	34	35	32	34	31	29	16%
20 min	0.89	0.68	0.6	0.51	0.38	0.34	43	41	39	40	37	44	17%
10 min	0.91	0.71	0.63	0.52	0.34	0.39	62	72	61	55	57	52	13%
05 min	0.97	0.74	0.72	0.47	0.44	0.22	99	95	93	75	111	70	6%
8 iterations													
50 min	0.87	0.70	0.63	0.58	0.52	0.53	76	82	84	77	78	82	9%
40 min	0.88	0.70	0.61	0.57	0.55	0.47	86	96	88	96	79	84	9%
30 min	0.88	0.73	0.62	0.62	0.50	0.52	100	114	99	110	89	78	11%
20 min	0.92	0.69	0.60	0.66	0.38	0.56	128	130	123	142	99	122	13%
10 min	0.92	0.68	0.60	0.57	0.33	0.71	181	216	190	156	130	192	13%
05 min	0.95	0.69	0.76	0.49	0.49	0.36	271	283	308	213	273	234	5%
*Cold phantom*													
2 iterations													
50 min	0.86	0.67	0.59	0.47	0.46	0.26	67	67	68	69	57	36	-
40 min	0.87	0.67	0.58	0.47	0.42	0.25	80	76	81	77	61	51	-
30 min	0.87	0.66	0.57	0.45	0.41	0.25	97	83	93	105	58	33	-
20 min	0.87	0.65	0.56	0.42	0.41	0.24	140	117	116	143	65	61	-
10 min	0.85	0.59	0.53	0.4	0.38	0.20	242	209	194	243	110	74	-
05 min	0.80	0.53	0.48	0.37	0.31	0.10	603	701	454	406	263	145	-
8 iterations													
50 min	0.90	0.71	0.62	0.51	0.52	0.32	475	442	545	266	214	144	-
40 min	0.91	0.70	0.62	0.51	0.47	0.30	584	632	495	226	227	156	-
30 min	0.90	0.69	0.61	0.49	0.45	0.29	703	630	666	329	240	141	-
20 min	0.89	0.68	0.59	0.44	0.46	0.27	1038	698	925	343	297	185	-
10 min	0.88	0.61	0.56	0.43	0.41	0.25	1577	1281	1490	597	399	321	-
05 min	0.83	0.55	0.51	0.39	0.34	0.12	3646	2384	1658	787	547	465	-

### Hot phantom

**Fig. 1:**
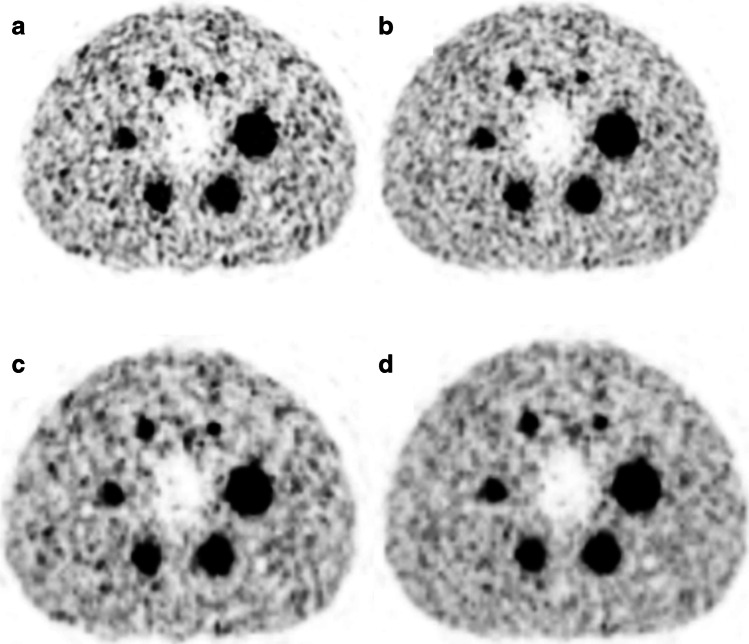
Axial slice of the NEMA hot phantom reconstructed with 440 × 440 (**a**, **b**) and 220 × 220 (**c**, **d**) reconstruction matrix, with HS (**a**, **c**) and UHS (**b**, **d**), with 4 iterations and 5 subsets, 2-mm FWHM Gaussian filter, and 50-min acquisition time

The lower matrix (220 × 220) exhibited similar RC_mean_ for the two largest spheres compared to the higher matrix (440 × 440) while higher values were present for the 4 smaller spheres (6 to 31%). In addition, HS provided significantly higher RC_mean_ values for the 4 smaller spheres (5 to 29%) compared to the UHS for all iterations setups. UHS provided higher SNR for both the lower and the higher reconstruction matrices compared to HS (220 × 220: 20 to 13% from the largest to the smallest sphere, 440 × 440: 23 to 15%). The lower matrix with UHS provided, respectively, 6 to 13% higher SNR compared to the higher matrix (Fig. 1). There was no difference between 2 and 4 iterations.

**Fig. 2:**
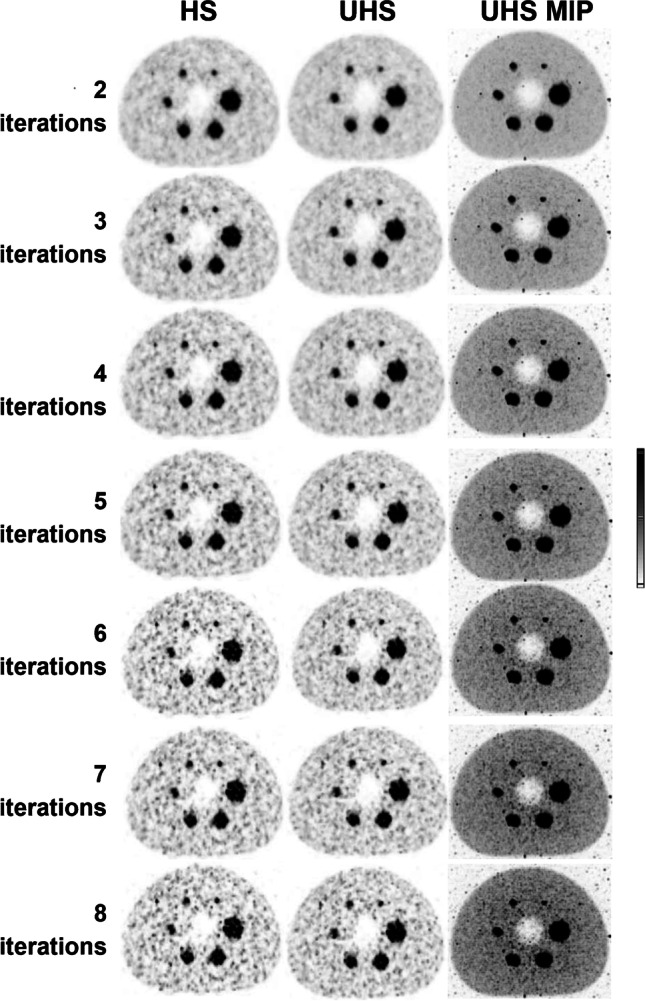
Axial slice of the NEMA hot phantom reconstructed with HS and UHS, with increasing number of iterations from 2 up to 8, with 2-mm Gaussian FWHM filter, and 50-min acquisition. The MIP is also shown. The noise level becomes more prominent from the 4 iterations and higher while UHS provides consistently a smoother background

For 2-mm FWHM Gaussian filter and 50-min acquisition the RC_mean_ was lowest for 2 iterations (0.84, 0.68, 0.63, 0.53, 0.45, and 0.36). Figure 2 shows axial slices of the hot phantom for all iterations and with the HS and UHS reconstruction modes as well as UHS maximum intensity projections (MIPs). The noise becomes more prominent with increasing number of iterations for both modes, especially from the 4 iteration and on. UHS however delivers higher SNR compared to the HS. On the MIPs, the noise distribution is better visualized on the background as well. The smallest sphere is discernible for any number of iterations.

**Fig. 3:**
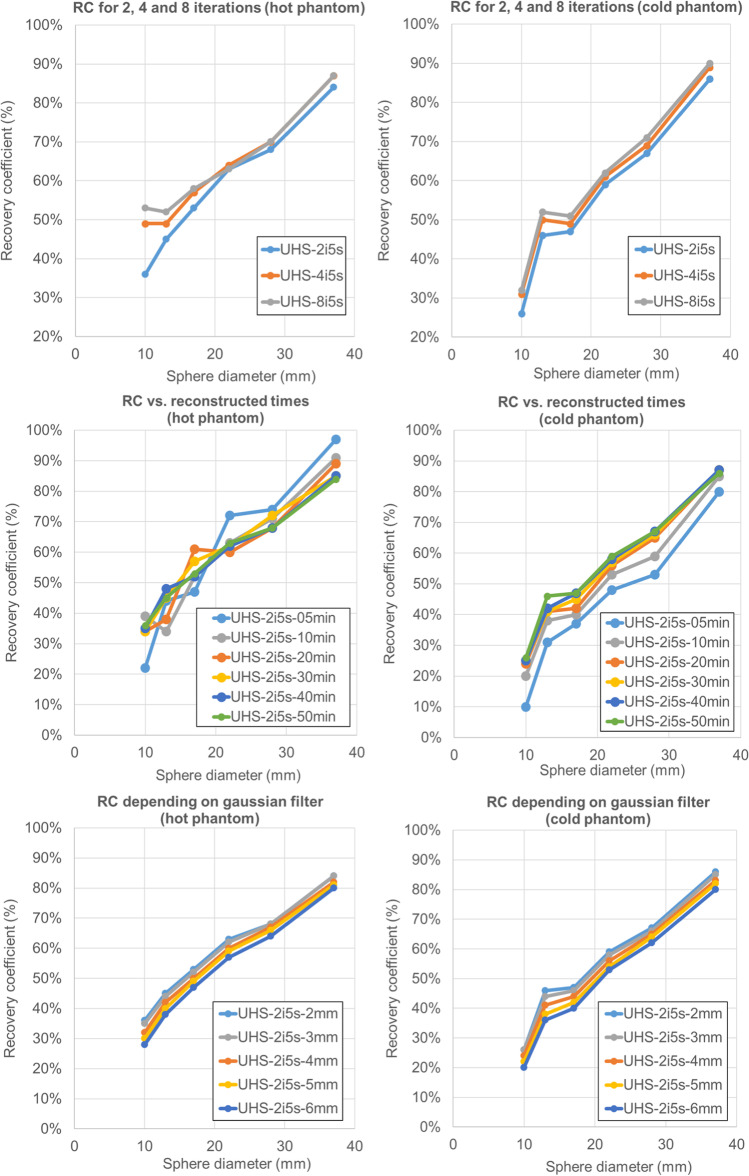
RC_mean_ curves for 2, 4, and 8 iterations, 50-, 40-, 30-, 20-, 10-, and 5-min reconstructions and Gaussian filter for both hot and cold phantoms

**Fig. 4:**
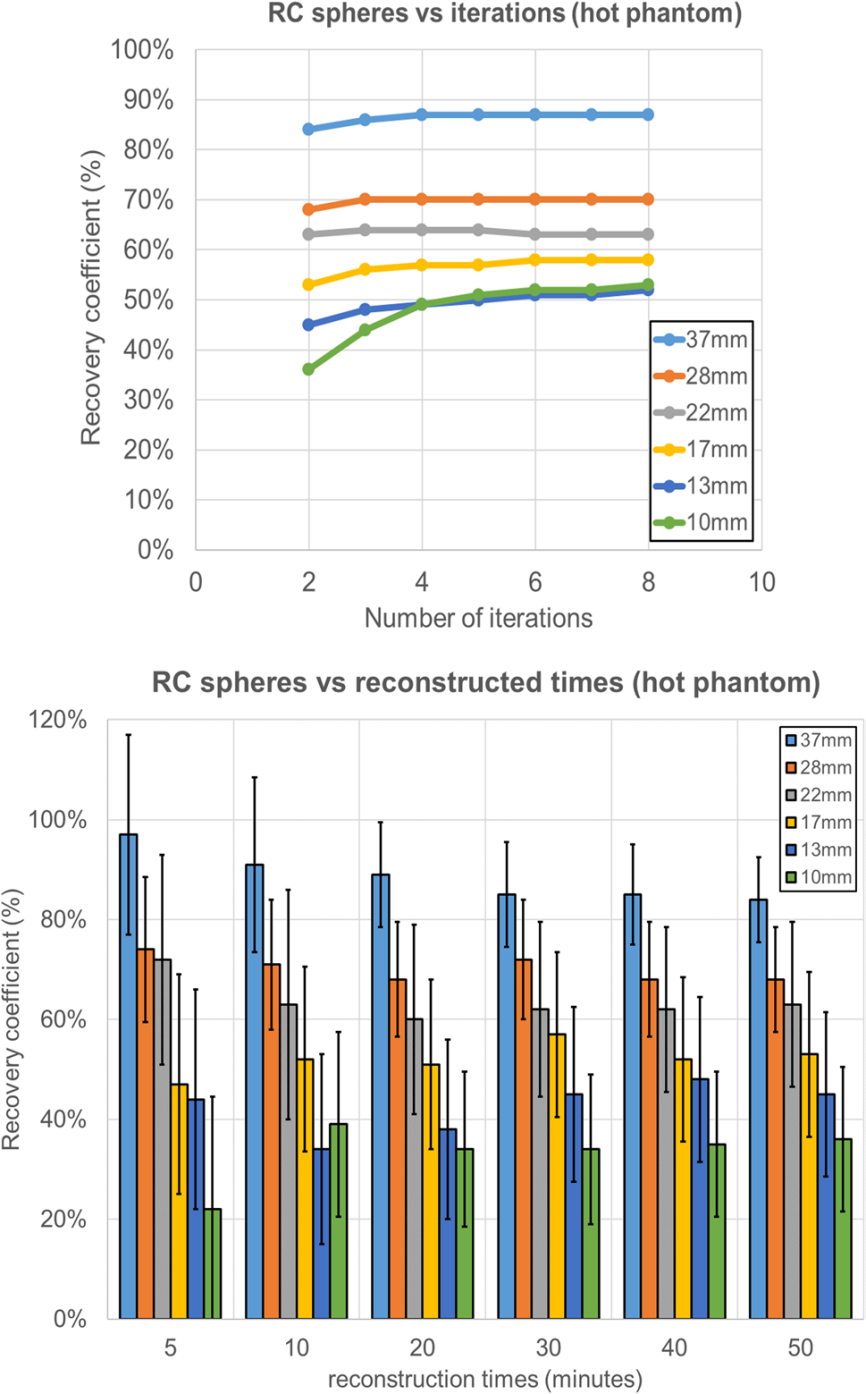
(top) RC_mean_ curves for all the spheres for a different number of iterations and (bottom) column chart of the RC_mean_ values for all the spheres with their measured errors against reconstructed times for the hot phantom

For the 5-min acquisition, the RC_mean_ for the spheres was 0.97, 0.74, 0.72, 0.47, 0.44, and 0.22 for 2 iterations. Figure 3 shows the RC_mean_ trend for all spheres against number of iterations, reconstructed times and Gaussian filter for both phantoms. It is worth mentioning that the drop in RC_mean_, while it is smoother for the hot phantom below the 17-mm sphere, is steeper for the cold phantom. Figure 4 shows that from 4 iterations, higher RC_mean_ values converge and reach a plateau for all spheres. In addition, RC_mean_ values are comparable from 50 min down to 20 min for all spheres and even down to 10 min for the bigger spheres.

**Fig. 5:**
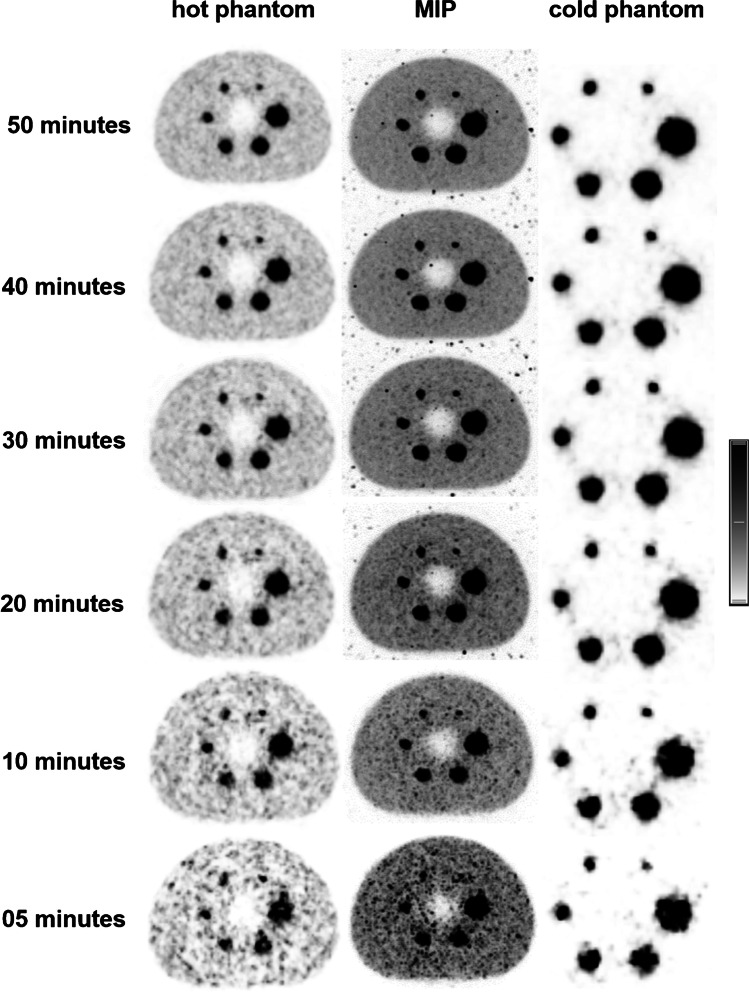
Axial slice of the NEMA hot and cold phantoms reconstructed with UHS, 2 iterations, 2-mm Gaussian FWHM filter, with the original acquisition time of 50 min and then with ever decreasing reconstructed times after rebinning of the list-mode data with 40, 30, 20, 10, and 5 min. The slice with the best visualization of the smallest hot sphere is shown along with the MIP. The smallest sphere is discernible even at 5 min

**Fig. 6:**
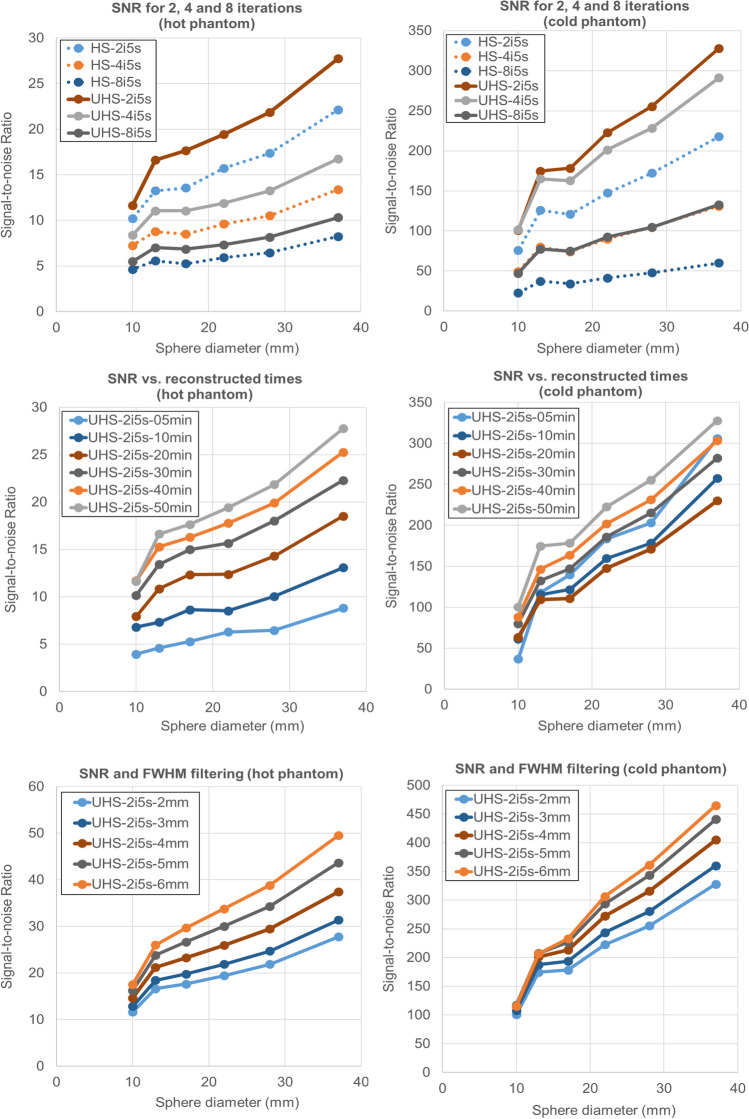
SNR curves for all the spheres for 2, 4, and 8 iterations, 50-, 40-, 30-, 20-, 10-, and 5-min reconstructions and Gaussian filter

The SNR was found to be highest for UHS 2 iterations, 50-min acquisition, with 6-mm FWHM Gaussian filter (49.51, 38.83, 33.76, 29.67, 26, and 17.57). Figure 5 shows axial slices for both phantoms and the MIPs for all reconstructed times from 50 down to 5 min. All spheres are visualized for all reconstructed times. The noise gets really prominent in the 5-min reconstruction. For 2-mm FWHM Gaussian filter, which is more clinically relevant due to sharper images, the SNR for 2 iterations was 27.76, 21.86, 19.42, 17.64, 16.62, and 11.65 (for HS 22.13, 17.36, 15.72, 13.57, 13.24, and 10.2). UHS provides 22.5%, 22.9%, 21.1%, 26.0%, 22.6%, and 13.2% increase in SNR compared to HS. The SNR for the UHS 20-min acquisition was 39.9%, 43.6%, 45%, 35%, 46%, and 44.4% less, than for the 50-min acquisition. The SNR diagrams can be found in Fig. 6.

The percent background variability for UHS 50-min acquisition was lowest for all the spheres, from the largest to the smallest, for the 2 iterations with 2-mm FWHM Gaussian filtering (27.59, 27.08, 27.36, 26.44, 30.11, and 33.51%). For the same parameters but with HS reconstruction, the percent background variability was 35.00, 36.05, 33.99, 33.41, 35.97, and 36.18% for 2 iterations. For the UHS 5-min acquisition, the percent background variability for 2 iterations and 2-mm FWHM Gaussian filter was 99.54, 95.72, 93.78, 75.5, 111.99, and 70.48%. Lung error was measured 18% (HS: 19%).

The RC_mean_ with 10 iterations and 1 subset for 5-min acquisition was 1.07, 0.88, 0.84, 0.46, 0.47, and 0.27 in contrast to 0.97, 0.74, 0.72, 0.47, 0.44, and 0.22 for 2 iterations for the biggest to the smallest sphere. Due to the longer convergence with 10 iterations, the RC_mean_ values were higher than with 2 iterations (except for the 17-mm sphere), and the difference was statistically significant (*p* < 0.05).

### Cold phantom

**Fig. 7:**
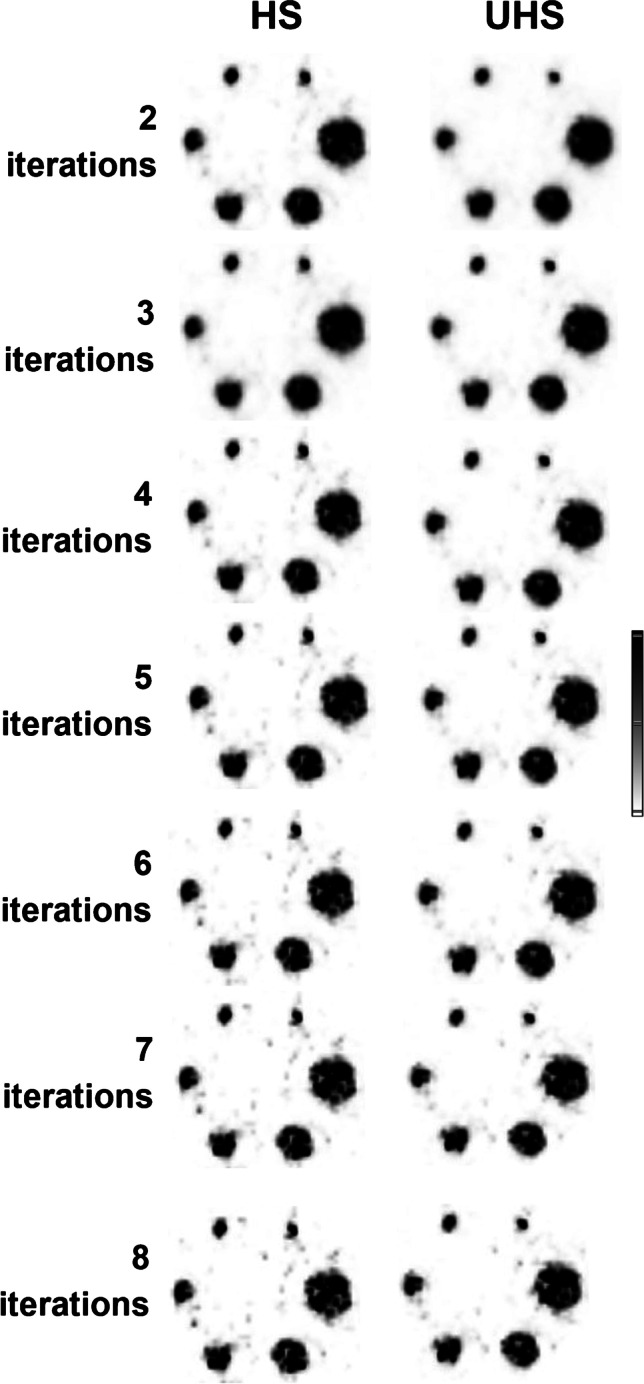
Axial slice of the NEMA cold phantom reconstructed with HS and UHS, with increasing number of iterations from 2 up to 8, with 2-mm Gaussian FWHM filter, and 50-min acquisition. The noise level becomes more apparent from the 4 iterations and higher while the UHS provides higher SNR especially between 2 and 4 iterations

The RC_mean_ was the lowest for 2 iterations and 50-min acquisition (0.86, 0.67, 0.59, 0.47, 0.46, and 0.26). Figure 7 shows the axial slices with HS and UHS for the cold phantom. Once again, the noise becomes higher after the 4 iteration, and all spheres are well visualized for all iteration numbers. The lower matrix with UHS provided higher SNR compared to the higher matrix (37 to 29% for the largest to the smallest sphere). UHS showed higher SNR to HS for both matrices (220 × 220: 55 to 51%, 440 × 440: 59 to 57%). For the 5-min acquisition, the RC_mean_ for the spheres was 0.80, 0.53, 0.48, 0.37, 0.31, and 0.10 for 2 iterations (Fig. 3).

The SNR was the highest for UHS 2 iterations, 50-min acquisition, with 6-mm FWHM Gaussian filter (564.00, 361.27, 306.54, 233.08, 206.83, and 115.37). For 2-mm FWHM Gaussian filter, which provides higher resolution, the SNR for 2 iterations was 327.86, 255.27, 222.68, 178.26, 174.60, and 100.47. The SNR for the 5-min acquisition was 305.84, 203.09, 183.55, 139.75, 117.83, and 36.89. For the HS mode, the SNR for 2 iterations, 50-min acquisition, and a 2-mm FWHM Gaussian filter was 217.62, 172.25, 147.47, 120.79, 125.80, and 75.78 (Fig. 6).

The percent background variability for UHS 50-min acquisition was the lowest for 2 iterations 27.59, 27.08, 27.36, 26.44, 30.11, and 33.51% (HS: 115.14, 123.05, 126.9, 78.78, 121.35, and 66.19%). For the UHS 5-min acquisition, it was measured 603.38, 701.46, 454.39, 406.59, 263.51, and 145.73%. Lung error was measured 120% for 2 iterations, 50 min with 2-mm FWHM Gaussian filtering (334% for 5-min reconstruction).

The 10 iterations and 1 subset produced the following RC_mean_ for a 5-min acquisition: 0.78, 0.54, 0.45, 0.4, 0.35, and 0.12 in contrast to 0.8, 0.53, 0.48, 0.37, 0.31, and 0.1 for the 2 iterations and 5 subsets for the biggest to the smallest sphere. The difference was not statistically significant (*p* < 0.05).

### Statistical analysis

UHS and HS did not present significant differences regarding the RC_mean_ values of the spheres, even though HC produced consistently higher RC_mean_ values to UHS. RC_mean_ values for all spheres did not exhibit significant differences between reconstructed times of 50 and 10 min for UHS 2 iterations (between 50 and 20 min for 8 iterations), while for the 5 bigger spheres, the RC_mean_ does not show significant differences between 2 and 4 iterations; 4 iterations provide significantly higher RC_mean_ for the smallest sphere for 20 min and below. The hot and cold phantoms presented similar RC_mean_ values for all spheres except for the smallest one where RC_mean_ was significantly higher in the hot phantom (0.36) than in the cold phantom (0.26).

### Patient data

**Fig. 8:**
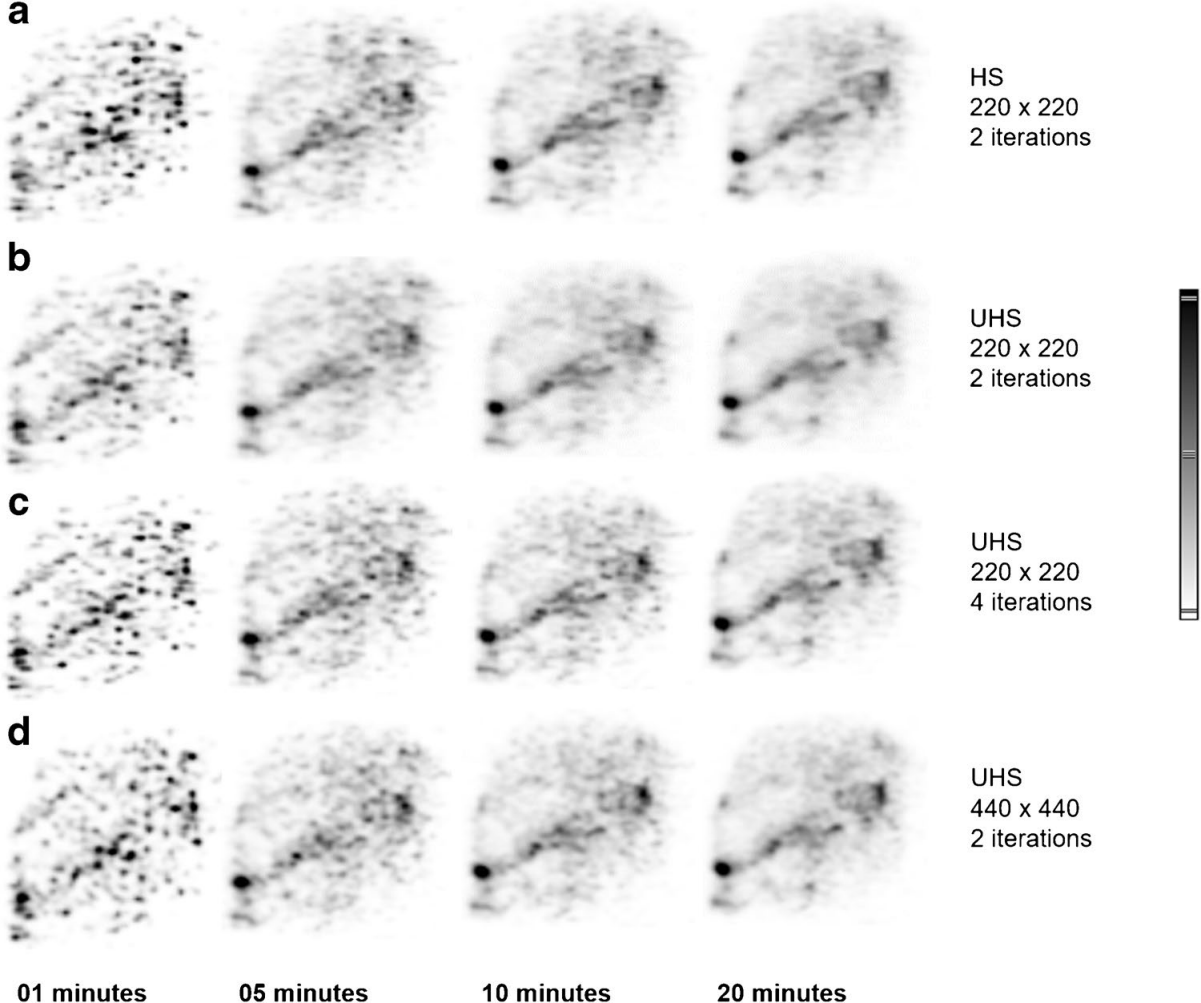
Coronal liver MIPs of a patient after.^90^Y radioembolization. Reconstructed images with 1-, 5-, 10-, and 20-min frames with a 220 × 220 matrix, 2 iterations, and HS (**a**), UHS (**b**), 4 iterations with UHS (**c**), and UHS 2 iterations with a 440 × 440 matrix (**d**)

As a convention, the values presented are in the following sequence: 20-, 10-, 5-, and 1-min reconstruction times. The SNR with UHS, for 2 iterations, was 19%, 20%, 24%, and 31% higher compared to 4 iterations (Fig. 8). The reconstruction with 2 iterations and UHS produced 2%, 10%, 17%, and 61% higher SNR compared to HS. Between reconstruction matrices of 220 × 220 and 440 × 440, the SNR percentage difference was lower than 10% for all reconstruction times for 2 iterations and UHS. The background liver VOI mean did not show any differences between 2 and 4 iterations for both UHS and HS. The COV was measured 6%, 15%, 16%, and 22% lower for UHS to HS for 2 iterations and 13%, 22%, 20%, and 26% for 4 iterations. The COV was 31%, 38%, 48%, and 64% lower for UHS 2 iterations compared to 4 iterations. The reconstruction with UHS 220 × 220 matrix exhibited 6%, 5%, 7%, and 16% lower COV compared to the 440 × 440 matrix.

## Discussion

In this study, the acquired NEMA body phantom datasets were reconstructed with different parameters like mode (UHS and HS), matrix (440 × 440 and 220 × 220), number of iterations (2–8), Gaussian FWHM filter (2–6 mm), and reconstructed times based on list-mode data rebinning (40, 30, 20, 10, and 5 min and the full acquired data of 50 min). The image quality was evaluated in terms of RC_mean_, background variability, lung error, and SNR. One patient dataset was reconstructed as well with 20-, 10-, 5-, and 1-min time frames and 2 iterations based on the evaluation of the phantom results.

The images reconstructed with a 220 × 220 matrix presented consistently a higher SNR (6% for the biggest sphere and 13% for the smallest sphere on average) than the images reconstructed with a 440 × 440 matrix, while the latter exhibited higher RC_mean_ values for all spheres.

In addition, the images with higher Gaussian FWHM filter resulted in higher SNR and higher loss of RC_mean_ values, as expected. UHS provided lower background variability and thus higher SNR (on average 5% for the largest sphere and 10% for the smallest sphere). The best reconstruction setup was deemed to be the 2 iterations, with 2-mm FWHM Gaussian filter. Twenty minutes seem to be the optimal acquisition time with 10 min seeming feasible as well, with minimal loss of SNR.

For the comparison of RC_mean_ values for various scanners below, some studies use the NEMA RC_mean_ definition while other follow the recommendations of the European Association of Nuclear Medicine (EANM) and EANM Research Ltd. (EARL). The RC_mean_ based on EARL is measured by drawing a VOI at 50% of the maximum pixel value corrected for background uptake [27]. Therefore, comparison may not be reliable since the NEMA takes into account all voxels of the VOI placed on a sphere, so it produces smoother RC_mean_ values. In any case, the EARL RC_mean_ values favor the other systems so the NEMA analysis described above provides conservative values.

A study comparing the Biograph mCT PET/CT (overall sensitivity 8.1 cps/kBq with an AFOV of 16.2 cm and 540 ps TOF [28]) and Biograph mMR PET/MR scanners with a 1:4 sphere to background ratio showed the highest RC_mean_ for the 37-mm sphere at 0.60 for mCT and slightly lower for mMR [29]. For the smallest sphere, 10 mm, the RC_mean_ was 0.20 for the mCT and slightly lower for the mMR. The reconstruction parameters used were as follows: 400 × 400 reconstruction matrix for mCT, 344 × 344 for mMR, 2 mm Gaussian FWHM filter, 3 iterations, and 21 subsets for both scanners, PSF TOF for the mCT, and only PSF for the mMR. The sphere activity concentration was around 1 MBq/ml.

Maughan et al. showed RC_mean_ values from 0.75 down to 0.30 with an 1:8 sphere to background activity concentration ratio, for the non-TOF Biograph mMR (overall sensitivity 15 cps/kBq [30]) with an AFOV of 25.6 cm [31]. The sphere activity concentration was 2.16 MB/ml, the scan was a 30-min single-bed acquisition, and the reconstruction parameters were PSF 3 iterations, 21 subsets with 5-mm FWHM Gaussian filter.

Kunnen et al. presented RC_mean_ values between 0.87 and 0.45 again with an 1:8 sphere to background activity concentration for Biograph Vision Edge 600 [32]. The scanner has an overall sensitivity of 16.4 cps/kBq and an AFOV of 26.1 cm [33]. The sphere concentration ratio was 2 MBq/ml, and the data were acquired during a 30-min two-bed acquisition. The reconstruction parameters were PSF TOF, 2 iterations, 5 subsets, no Gaussian post-filter, and a reconstruction matrix of 220 × 220. The RC_mean_ definition followed the NEMA standard.

Scott et al. evaluated the GE Discovery 710 PET/CT (General Electric, WA, USA) scanner with an overall sensitivity 7.25 cps/kBq and an AFOV of 15 cm [34] with an 1:8 sphere to background activity concentration ratio that provided the highest RC_mean_ of 0.73 for the 37-mm sphere and 0.39 for the 17-mm sphere, for a 30-min two-bed acquisition [35]. The two smallest spheres (13 mm and 10 mm) were not taken into consideration due to high uncertainties in the measurements. In this study, the reconstruction used a Bayesian penalized likelihood reconstruction algorithm (Q. Clear, GE Healthcare) in full convergence so iterations and subsets do not apply.

Another study with the Signa PET/MR (General Electric, WA, USA), with overall sensitivity of 21 cps/kBq and an AFOV of 25 cm [36], showed RC_mean_ values ranging from 0.80 down to 0.27 with a 1:4 sphere to background ratio, 0.75 MBq/ml sphere concentration, a 60-min single-bed acquisition, and images reconstructed with TOF2 iterations, 28 subsets, and 4-mm FWHM Gaussian filter [37]. The Biograph Vision Quadra shows improved contrast recovery coefficients for the smaller spheres, compared to PET/CT systems with standard AFOV. The smallest sphere is clearly visible even in the 5-min scan, although at the expense of increased noise, suggesting that scans shorter than 20 min could be clinically feasible without loss of diagnostic accuracy of small lesions. Some of the scanners with standard AFOV could not discern the smallest sphere at all or could only depict them with an acquisition time of at least 30 min and at least double activity concentration than in the study described here. Another good point to note here is that the other studies used at least an 1:8 sphere to background activity concentration while the study with the Biograph Vision Quadra used even less at 1:10 that could further have an effect on lowering the RC_mean_ values for all spheres.

For the patient data UHS delivers better image quality with higher SNR at the low count statistics of the 5-min acquisition compared to HS while it delivers a mediocre improvement with 20-min acquisition. Reconstruction with 2 iterations provides improved image quality with lower noise and higher SNR compared to the standard protocol with 4 iterations. In general, after 4 iterations, noise levels become more prominent. For post-treatment dosimetry validation where quantification of lesions is essential, 4 iterations could be the better reconstruction parameter since it can deliver higher recovery coefficients, but 2 iterations could be better for small lesion detectability. However, in this study, we prioritized the SNR, this is why we reconstructed the patient data also with 2 iterations. A future study that would also evaluate the quantification and dosimetric quantities could establish the optimal trade-off of reconstruction parameters between the SNR and RC_mean_. Furthermore, it seems that based on the size of the spheres, the reconstruction parameter, like number of iterations that delivers improved SNR and RC_mean_, varies. This is in accordance with another phantom study that used a digital photon counting PET for optimizing reconstruction parameters [38] and showed that depending on the size of the sphere a different number of iterations and subsets should be used for more accurate quantification [38]. For the smallest spheres, for example, a number of iterations even higher than 4 could be justified as long as noise level is acceptable. In the end, the image quality depends on the density of the patient and the injected activity of ^90^Y.

The smallest sphere was better visible in the hot phantom compared to the cold phantom. This could be explained because the background activity in the hot phantom minimizes the partial volume effect in uptake areas near the spatial resolution limit. The impact of the different reconstruction parameters was the same for both phantoms.

The optimal reconstruction protocol to maximize the SNR, based on the phantom-based evaluation, appears to be 2 iterations with 2-mm FWHM Gaussian filter, with a reconstruction matrix of 220 × 220 and using the UHS mode. The acquisition time with 20 min delivers high SNR and resolution images, comparable to the 50-min acquisition. The results show that 10 min seems to be clinically feasible as well, even though noise starts to become more apparent, but more testing with clinical data is required.

Except for the standardized analysis based on RC_mean_ or SNR on optimizing the reconstruction parameters for ^90^Y post-treatment dosimetry validation, another approach is to evaluate the dose volume histograms (DVH) of the spheres of a NEMA body phantom. A study has already used this method and showed that by minimizing the errors in DVH, joint optimization of iterations and filtering for ^90^Y volumetric quantification can be achieved [39]. The DVH method was used also to evaluate patient data for pre-treatment dosimetry plan based on Technetium-99 m (^99m^Tc) macroaggregated albumin (MAA) SPECT/CT [40].

PET can provide images with higher resolution and more accurate quantification compared to SPECT, thus setting itself a better candidate imaging method for post-treatment dosimetry evaluation. In particular, the LAFOV PET/CT could further boost the involvement of PET in ^90^Y post-treatment clinical practice by providing quantitative images with high SNR at reduced scan times, improving the whole patient throughput. As expected, the applications of the Biograph Vision Quadra in ^90^Y SIRT imaging benefits from the combination of increased sensitivity due to LAFOV and the noise reduction due to very good time resolution (about 230 ps).

In conclusion, the NEMA phantom-based evaluation for ^90^Y imaging with the Biograph Vision Quadra provides images with higher recovery coefficient values for the smallest spheres than any other commercial PET/CT with standard AFOV as well as high SNR for all spheres at significantly reduced scanned times. Specifically, the reconstruction with UHS, 2 iterations, 2-mm FWHM Gaussian filter, and a reconstruction matrix of 220 × 220 seems to provide the highest SNR while 4 iterations and higher could be optimal for small lesion quantification for post-treatment ^90^Y validation, given however that the increased noise level that comes with higher number of iterations is acceptable. Scan times of only 10 min provide images with an acceptable SNR loss, but more testing for clinical validation is required.
